# A sigh-performance hydrogen gas sensor based on Ag/Pd nanoparticle-functionalized ZnO nanoplates†

**DOI:** 10.1039/d3ra01436c

**Published:** 2023-04-26

**Authors:** To Thi Nguyet, Dang Thi Thanh Le, Nguyen Van Duy, Chu Thi Xuan, Sven Ingebrandt, Xuan Thang Vu, Nguyen Duc Hoa

**Affiliations:** a International Training Institute for Materials Science (ITIMS), Hanoi University of Science and Technology (HUST) No 1 Dai Co Viet, Hai Ba Trung Ha Noi Vietnam ndhoa@itims.edu.vn hoa.nguyenduc@hust.edu.vn; b Institute of Materials in Electrical Engineering 1, RWTH Aachen University Sommerfeldstr. 24 Aachen 52074 Germany

## Abstract

As a source of clean energy, hydrogen (H_2_) is a promising alternative to fossil fuels in reducing the carbon footprint. However, due to the highly explosive nature of H_2_, developing a high-performance sensor for real-time detection of H_2_ gas at low concentration is essential. Here, we demonstrated the H_2_ gas sensing performance of Ag/Pd nanoparticle-functionalized ZnO nanoplates. Bimetallic Ag/Pd nanoparticles with an average size of 8 nm were prepared and decorated on the surface of ZnO nanoplates to enhance the H_2_ gas sensing performance. Compared with pristine ZnO, the sensor based on ZnO nanoplate doped with Ag/Pd (0.025 wt%) exhibited an outstanding response upon exposure to H_2_ gas (*R*_a_/*R*_g_ = 78 for 500 ppm) with fast response time and speedy recovery. The sensor also showed excellent selectivity for the detection of H_2_ over the interfering gases (*i.e.*, CO, NH_3_, H_2_S, and VOCs). The superior gas sensing of the sensor was dominated by the morphological structure of ZnO, and the synergistic effect of strong adsorption and the optimum catalytic characteristics of the bimetallic Ag/Pd enhances the hydrogen response of the sensors. Thus, bimetallic Ag/Pd-doped ZnO is a promising sensing material for the quantitative determination of H_2_ concentration towards industrial applications.

## Abstract 2

The Ag/Pd–ZnO nanoplates exhibit 47 folds enhancement response to hydrogen gas. This demonstrates the catalyst phenomenon of Ag/Pd nanoparticles on the hydrogen sensing performance.

## Graphical abstract

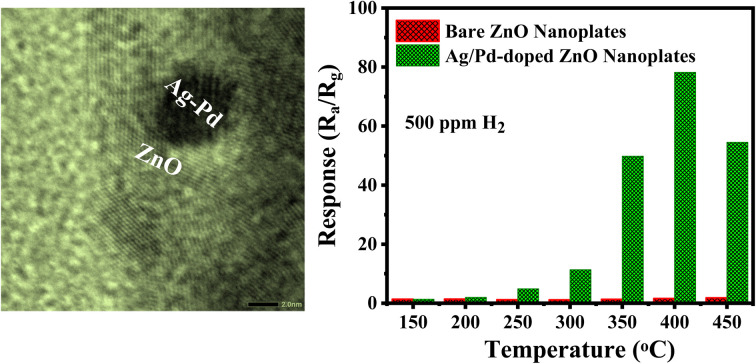

## 1. Introduction

The reduction of the carbon footprint by using green hydrogen (H_2_) fuel cells for renewable, sustainable, and clean energy has attracted great attention.^1^ H_2_ fuel cells have been extensively applied in start-of-the-art devices, such as smartphones, fuel cell vehicles, electronic devices, and military vehicles.^2^ However, ensuring the safe utilization of H_2_ is a great issue, because H_2_ is highly flammable and explosive, especially in concentrations of 4–75% (by volume in air).^3^ The H_2_ molecule is ultrasmall in size (0.289 nm), has a very high diffusion coefficient of ∼0.6 cm^2^ s^−1^, and therefore facilitates its leakage from containment cells during storage and transportation.^3^ In addition, H_2_ gas is almost tasteless, odorless, and colorless, leading to undetected H_2_ by human eyes and other senses.^4^ Therefore, the development of an effective device for detecting and/or alarming hydrogen leakage during storage and transportation is crucial. Real-time H_2_ monitoring devices are required for the precise warning for H_2_ concentrations from tens of part per million (ppm) to hundreds of ppm.

In the scientific literature, there are many approaches, such as the use of electrochemical,^5^ optical,^6^ acoustic,^7^ and resistive sensors,^8^ that have been applied to monitor H_2_ gas. Among these techniques, the resistive sensor is an attractive device for real-time gas monitoring because of its compact size, low cost, operating stability over longer periods, and ease of use in practical circumstances.^9^ Semiconducting metal oxides (SMOs), such as zinc oxide (ZnO), tin(iv) oxide (SnO_2_), and indium oxide (In_2_O_3_), with different morphologies have been used as gas sensing layers because of their good sensing capabilities such as low detection limit, high sensitivity with fast response and recovery time, and can be integrated into portable devices.^10^ As an n-type semiconductor with a wide band gap (3.7 eV), ZnO is an appropriate material for gas sensors because of its known superior sensing properties and its facile manufacturing technology.^11^ Various nanostructures of ZnO (*i.e.*, nano lily-bud garden,^12^ nanoparticles,^13^ nanowires,^14^ and nanorods^15^) have been used as sensing materials in gas sensors. Among the different morphologies, ZnO nanomaterial has been extensively studied by enhancing the gas adsorption rate and electron transport on an ultrathin surface, making it capable to sense various gases.^16,17^ For instance, porous ZnO nanosheet with a thickness of 80 nm has been reported to exhibit the best response to NO_2_ gas at 200 °C.^16^ The ZnO nanosheet sensor reached a response value of 175.5 toward 100 ppm of tetraethylammonium.^17^ Some other hazardous gases (*i.e.*, ethanol, CO, NO_2_, and H_2_S) could also be detected by the ZnO nanomaterial.^18,19^ Recently, ZnO nanostructures have also been used to detect H_2_ gas leaks, where the ZnO nanorods exhibited a low response of 9.2 to 1000 ppm of H_2_ at 225 °C.^20^ However, pure ZnO-based sensors showed low sensitivity and poor selectivity. Thus, many significant studies have been performed to overcome these limitations.^21^

One of the techniques that can remarkably improve the four factor “S” sensitivity, selectivity, stability, and response speeds of gas sensors is the use of noble metals to functionalize the surface of a metal oxide by utilizing the spillover effect and unique catalytic property of these nanoparticles.^19–21^ For instance, Kim *et al.*^22^ synthesized Pd-decorated ultrathin ZnO nanosheets in detecting hydrogen in a gaseous mixture with benzene. ZnO thin film decorated with Pt nanoparticles was used to detect H_2_ gas in the mixture with humidity.^21^ Agarwal *et al.*^23^ used Ag/ZnO hollow tube as sensing material to enhance the H_2_ gas sensing performance. Ag catalyst nanoparticles were functionalized on the metal–organic framework-derived ZnO hollow nanocages.^24^ However, the use of single-metal material as catalyst in enhancing the gas sensing performance have some limitations. For instance, when operating at high temperature, metal catalysts undergo oxidation, leading to reduced catalytic activity and deterioration of sensing performance.^25^ Alloying nanoparticles with two or more metal elements have recently been reported to show superior sensing properties in terms of electronic and geometric parameters compared with monometal doping catalyst for gas sensors.^26,27^ The alloy of Pt/Pd ultrathin film has been deposited on the surface of ZnO nanorods to enhance H_2_ gas sensitivity.^28^ Le *et al.*^29^ reported efficient H_2_ gas sensor by using PdAu@ZnO core–shell nanostructure. However, the PdAu nanoparticles were prepared by a sodium citrate reduction method with CTAB as surfactant, which may cause contamination of the sensing materials. Zamora Zeledón *et al.* have recently reported enhanced electrocatalyst approach by tuning the electronic structure of Ag/Pd alloys for reducing alkaline oxygen but not sensing application.^30^ Given that silver (Ag) is much cheaper (by 110-folds) than Au or Pt, using the Ag/Pd alloy as the catalyst is more economical. The use of Ag/Pd alloy also utilize the synergic catalytic effect of both metals.^29^ Furthermore, the alloy is thermal stable over oxidation, maintaining its catalytic activity thus enable the long-term stability of the sensor. However, few studies have used Pd/Ag nanoparticle-decorated ZnO nanoplates to enhance the H_2_ gas sensing performance.^31^

Here, Ag/Pd nanoparticles were uniformly functionalized on the surface of hydrothermally grown ZnO nanoplates to enhance H_2_ gas sensing properties. The Ag/Pd–ZnO nanoplate sensors were fabricated by simple and straightforward drop-casting onto interdigital electrodes, which were previously patterned using photolithography and a sputtering approach, followed by an annealing treatment at 600 °C. The resistive-type gas sensors were tested for hydrogen detection and showed sensing properties with sensitivity and selectivity to other gases (*i.e.*, ethanol, methanol, isopropanol, triethylamine, NH_3_, CO, and H_2_S). The device could detect low concentration of H_2_ from 25 ppm to 500 ppm with ultrafast response and recovery times. The role of the Ag/Pd nanoparticle decoration in the enhancement of the gas sensing performance of ZnO was also discussed intensively.

## 2. Experimental

In a typical process, the analytical substances consisting of zinc sulfate heptahydrate (ZnSO_4_·7H_2_O), urea (CH_4_N_2_O), silver nitrate (AgNO_3_), palladium(ii) chloride (PdCl_2_), glycerol, ethylene glycol (EG), and poly(vinyl pyrrolidone) (PVP) were used as precursors. All chemicals were of analytical grade and used without any further purification.

### 2.1. Preparation of ultrathin ZnO porous nanoplates

**Fig. 1:**
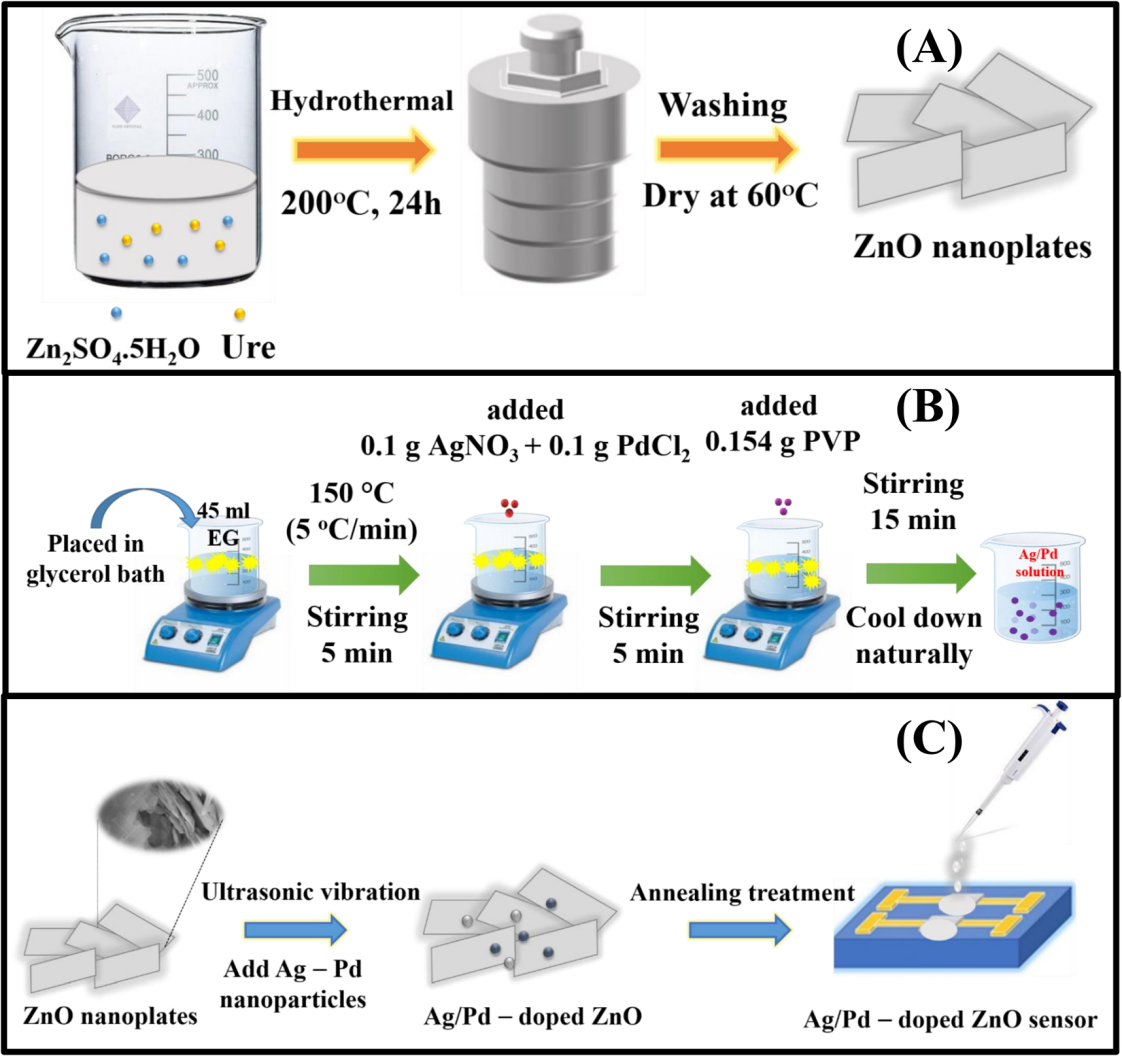
(A) Schematic diagram of the synthesis of pristine ZnO nanoplate, (B) Ag/Pd-doped ZnO nanoplates, (C) and the gas sensor fabricated by the drop-casting process.

The ZnO nanoplates were prepared according to a previous report.^32^ ZnSO_4_·7H_2_O (10 mmol) was dissolved in 30 mL of deionized water, and 20 mmol urea solution was added with further stirring for 15 min. The resulting solution was directly transferred into a Teflon-lined stainless-steel autoclave. The autoclave was sealed and loaded in an electric oven for hydrothermal growth at 200 °C for 24 h. After being naturally cooled to room temperature, the product was washed several times with deionized water and ethanol solvent by centrifugation at 4000 rpm for 20 min. The obtained white precipitate was dried in an electrical oven at 60 °C for 24 h. The ZnO nanoplates were annealed at 600 °C for 2 h in an airflow to reduce the defects and obtain a high crystal quality (Fig. 1A).

### 2.2. Synthesis of bimetallic Ag/Pd nanoparticles

The bimetallic Ag/Pd nanoparticles were prepared using a modified polyol technique as shown in Fig. 1B.^33^ EG was heated in a three-necked flask containing glycerol up to 150 °C with a heating rate of 5 °C min^−1^. The solution was held for 5 min. Then, 0.1 g of AgNO_3_ and 0.1 g of PdCl_2_ were added, and the mixture was continuously stirred for 5 min. Afterward, 0.15 g of PVP was injected into the mixture and further stirred for 15 min to reduce Pd^2+^ and Ag^+^ into Ag/Pd nanoparticles. Finally, the colloidal Ag/Pd solution was cooled gradually to room temperature. The obtained Ag/Pd colloidal were stored inside a refrigerator until usage.

### 2.3. Preparation of Ag/Pd–ZnO nanoplates and fabrication of gas sensor

The Ag/Pd–ZnO nanoplate-based gas sensors were prepared by a drop-casting method according to the literature,^34^ as shown in Fig. 1C. Typically, 100 mg of the collected ZnO nanoplate powders and Ag/Pd nanoparticles were dispersed ultrasonically in ethanol solvent for 30 min to form a colloidal solution. Here, the ZnO nanoplates were mixed with Ag/Pd nanoparticles (0.0125 wt%, 0.025 wt%, and 0.05 wt%). The Ag/Pd–ZnO nanoplate colloidal solution was then dropped on the Pt interdigital electrodes deposited on thermally oxidized silicon substrate to form thick film sensors. The fabricated sensors were annealed at 600 °C for 2 h to increase stability and reduce the contact resistance between the sensing material and the Pt electrodes.

The fabricated sensors were characterized *via* a laboratory-made gas sensing measurement system.^35^ H_2_ (10.000 ppm) diluted in N_2_ was used as the standard test gas. A series of mass flow controllers were used to mix the standard gas with dry air to achieve the desired gas concentrations ranging from 25 ppm to 500 ppm. Prior to the measurements, the sensor was preheated at 600 °C for 2 h to stabilize the contact between the Pt electrode and Ag/Pd–ZnO nanoplates. The gas concentration was controlled by modifying the flow rate of air and standard gas, but the total flow rate introduced in the measuring chamber was fixed at 400 sccm. The sensor resistance *versus* testing time was recorded using a source measurement unit instrument (Keithley 2602B). The response value of the sensor was determined by the ratio of *R*_a_/*R*_g_ toward reducing gases or *R*_g_/*R*_a_ in the case of oxidizing gas, where *R*_a_ and *R*_g_ were the resistances of the sensor in air and target gas, respectively. The response time (*τ*_res_) and recovery time (*τ*_rec_) were determined as the times to reach 90% saturation of the response after exposure to the analyte gas and air, respectively. The detection limit (DL) was calculated using the slope of the linear regime as described before.^36^

The structure, morphology, and composition of the prepared materials were characterized using a scanning electron microscope (SEM, JEOL 7600F), energy-dispersive X-ray spectroscope (EDS), X-ray diffractometer (XRD, D2 Phaser, Bruker) with Cu K_α_ radiation, and high-resolution transmission electron microscope (HRTEM, JSM 2100 F).

## 3. Results and discussion

### 3.1. Characterization of materials

**Fig. 2:**
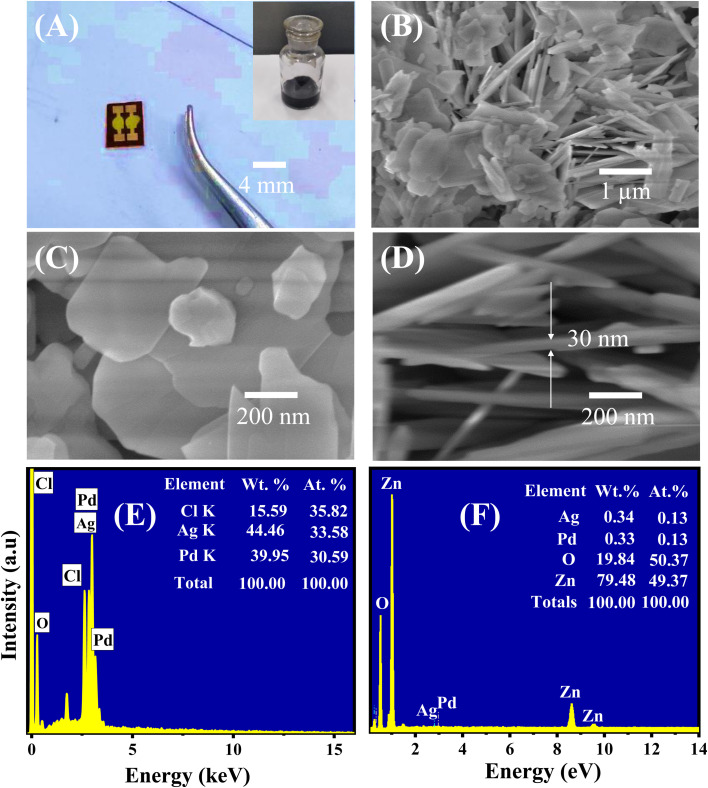
Photo of the (A) fabricated 0.025 wt% sensors, and inset is a photo of the sensing material solution; and SEM images of the Ag/Pd-doped ZnO nanoplates at (B) low magnification, (C) high magnification, and (D) cross-sectional view. EDS spectra of the (E) Ag/Pd nanoparticle and (F) Ag/Pd-doped ZnO nanoplates.

A photograph of the fabricated 0.025 wt% sensors is shown in Fig. 2A, where the inset is the sensing material solution. The morphology of the as-synthesized ZnO nanoplates modified with Ag/Pd nanoparticles was investigated using the SEM, as shown in Fig. 2A and B. The low-magnification SEM image (Fig. 2A) demonstrates that the ultrathin nanoplates which were stacked together and oriented randomly. The high-magnification SEM image shows the nanoplates with an average diameter of 500 nm (Fig. 2B). The cross-sectional SEM image confirmed that the ZnO nanoplates were ∼30 nm in thickness (Fig. 2D). The BET surface area of the ZnO nanoplates was ∼75 m^2^ g^−1^ (data not shown). The large surface area and thin thickness facilitated the sensing properties of the ZnO nanoplates, which was revealed in the previous study.^37^ The existence of the Ag/Pd nanoparticles was difficult to observe from the SEM images. The Ag/Pd nanoparticles were possibly decorated on the top and edge of the ZnO nanoplates but were too small to be visualized from the SEM images. This inference would be confirmed using the high-resolution TEM (HR-TEM) images in the following section. To evaluate the elemental composition of the Ag/Pd nanoparticles and the as-prepared Ag/Pd-doped ZnO nanoplates, EDS analyses were conducted with electron beam of 15 keV. The corresponding results are shown in Fig. 2E and F. The chemical compositions are displayed comprehensively in the insets. Three elements, namely, Ag, Pd, and Cl, were found in the Ag/Pd nanoparticles (Fig. 2E). The Cl peak was observed in the EDS spectra of the bimetallic Ag/Pd sample, depicting the Cl^−^ ion contamination from the PdCl_2_ precursor. For the Ag/Pd–ZnO sample (Fig. 2F), the EDS results showed that the sample had Zn, O, Ag, and Pd at 78.48 wt%, 19.84 wt%, 0.33 wt%, and 0.34 wt%, respectively. Thus, the atom ratio of Ag and Pd was 1 : 1, or the alloy Pd_0.5_Ag_0.5_ and ZnO_1+*δ*_ were obtained. The EDS mapping result also demonstrated the well-defined dispersion of Pd_0.5_Ag_0.5_ nanoparticles on the surface of ZnO nanoplates (Fig. S1 ESI†).

**Fig. 3:**
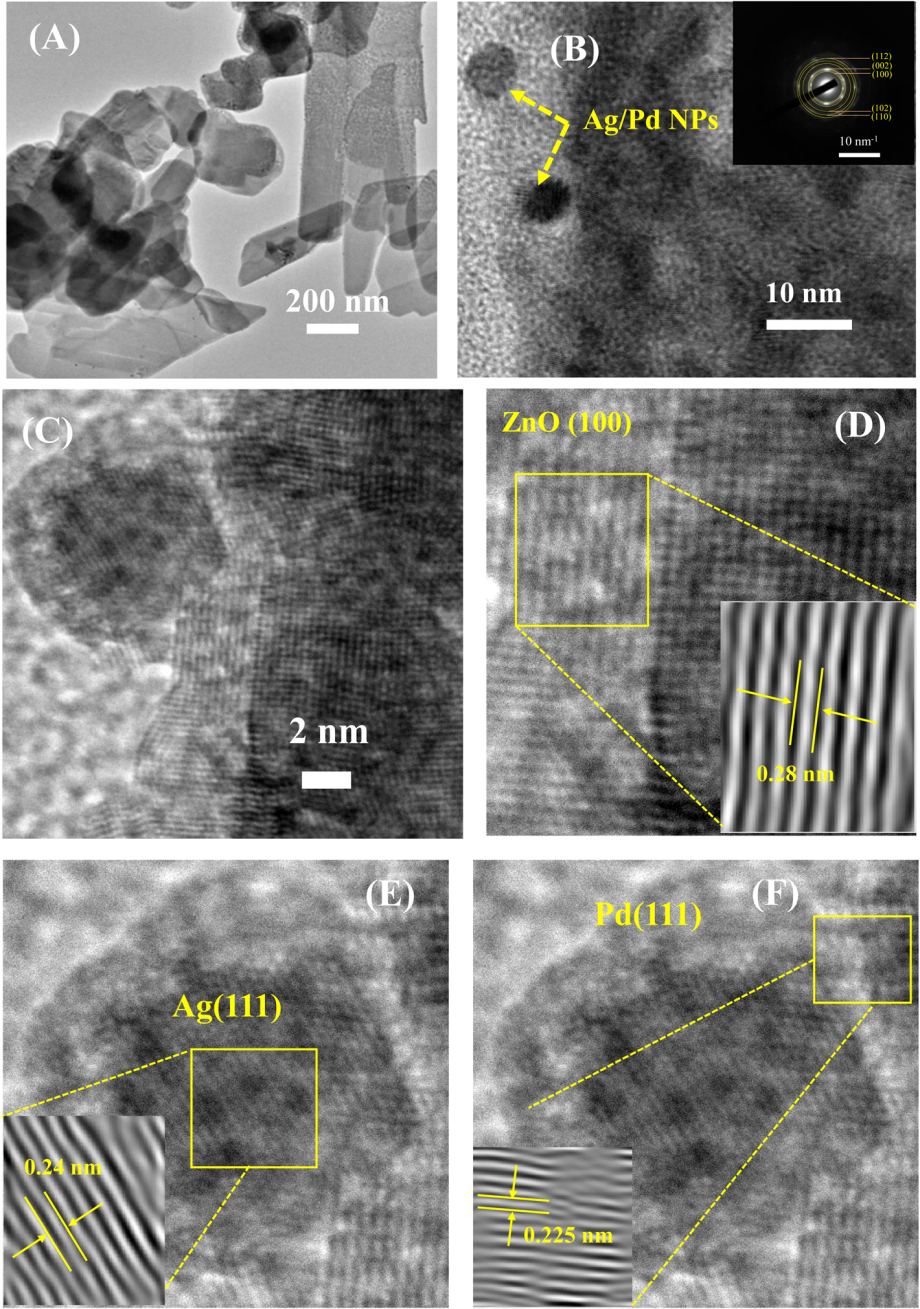
(A) Low and (B–F) high magnification TEM images of the 0.025 wt% Ag/Pd-doped ZnO nanoplates. Inset (B) shows the corresponding selected area electron diffraction.

The TEM and HRTEM analyses were employed to further study the nanostructure and presence of Ag/Pd nanoparticles on the surface of the ZnO nanoplates. The TEM micrographs in Fig. 3A revealed the plate-like features of the ZnO nanoplates with different shapes. Fig. 3B also shows the existence of the Ag/Pd nanoparticles as black dots with small size of eight nm and distributed randomly on the surface and at the edge of the ZnO nanoplates. The HRTEM micrographs of the prepared sample shown in Fig. 3B and C confirmed that the ZnO nanoplate was a polycrystal, while the Ag/Pd nanoparticle was a single crystal. As shown in the inset of Fig. 3B, the corresponding selected area electron diffraction patterns of the Ag/Pd-doped ZnO nanoplates confirmed that the bright rings showed the nature of the ZnO polycrystals. A zoomed out HRTEM image of the Ag/Pd-doped ZnO sample established the coexistence of Ag/Pd and ZnO nanocrystals with interplanar spacing of 0.28 nm (Fig. 3D), 0.23 nm (Fig. 3E), and 0.225 nm (Fig. 3F), corresponding to the lattice fringes of (100) planes of hexagonal ZnO, (111) planes of Ag, and face-centered cubic Pd.^38,39^ Thus, the HRTEM images confirmed the successful decoration of the surface of ZnO nanoplates with bimetallic Ag/Pd nanoparticles.

**Fig. 4:**
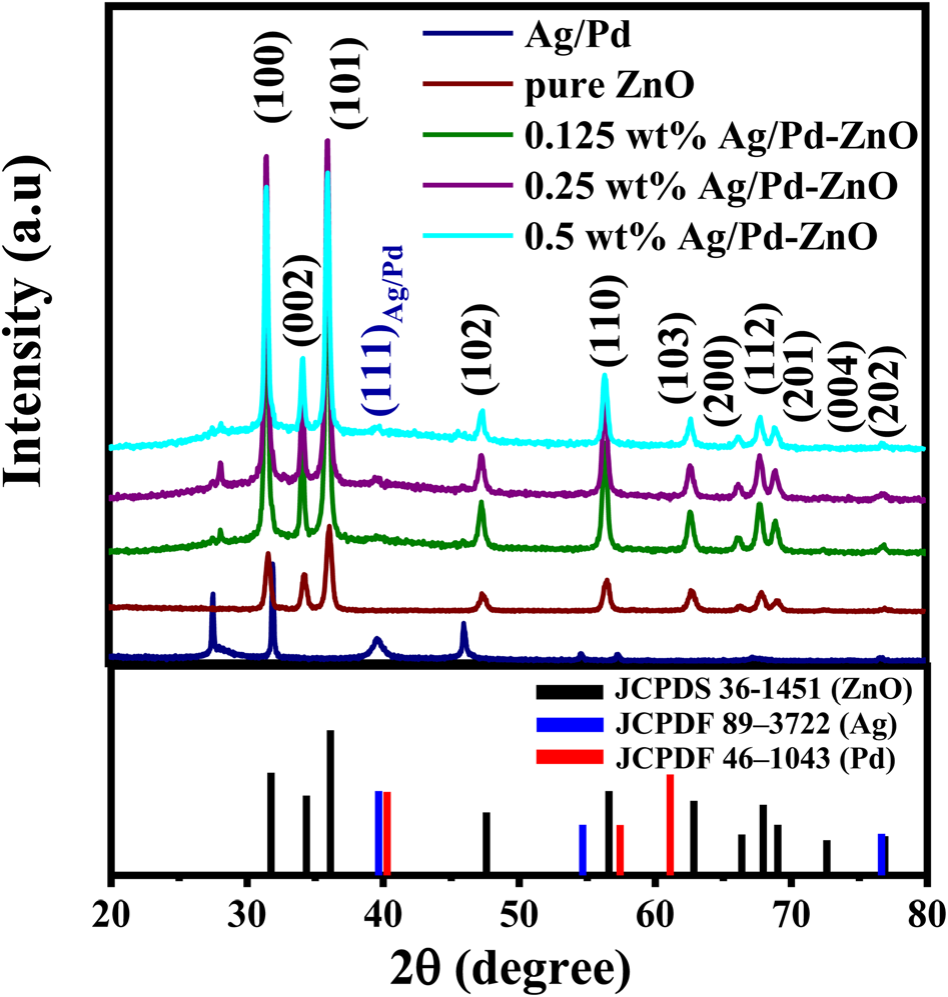
XRD patterns of the bimetallic AgPd, pristine ZnO nanoplates, and Ag/Pd-doped ZnO nanoplates.

The effect of incorporating Ag/Pd nanoparticles on the microstructure and crystalline features of the ZnO nanoplates was also investigated by X-ray diffraction. As shown in Fig. 4, typical diffraction peaks of the pure ZnO nanoplates after annealing at 600 °C in 2 h were indexed to the wurtzite (hexagonal) phase of ZnO (JCPDS 36-145) without significant impurity peak. The major peaks were located at 2*θ* of 31.75°, 34.35°, 36.12°, 47.59°, 56.59°, 62.83°, 66.36°, 67.93°, 69.0°, 72.61°, and 76.86°, corresponding to the (100), (002), (101), (102), (110), (103), (200), (112), (201), and (202) crystal planes.^40^ The distinct peak indicated the successful preparation of single-phase ZnO nanoplates. The XRD pattern of the Ag/Pd nanoparticles dispersed on the Si substrate with thermal treatment at 150 °C for 2 h is shown in Fig. 4. The diffraction peaks of the face-center cubic Ag and Pd were observed at 2*θ* of 39.67°, 40.28°, 46.06°, 65.25°, 67.30°, and 76.65° indexed to (111)_Ag_, (111)_Pd_, (002)_Pd_, (220)_Ag_, (220)_Pd_, and (311)_Ag_ crystallographic planes of Ag (JCPDF 89-3722) and Pd (JCPDF 46-1043).^41^ Here, the (111) peak of Ag/Pd nanoparticles lines between the (111) peaks of face-centered cubic crystals Ag and Pd, indicating the successful formation of the Ag/Pd alloy.^30^ The well-defined diffraction peaks indicated that the Ag/Pd alloy was formed as a solid solution. Meanwhile, the Ag/Pd-doped ZnO exhibited additional peaks of Ag/Pd along with the typical diffractions of hexagonal ZnO crystal. All additional peaks detected in the XRD analyses indicated the formation of the alloying metal Ag/Pd nanoparticles on the surface of ZnO nanocrystals. Using the Scherrer formula, the crystal size of the ZnO nanoplates can be calculated to about 21.44 nm,^42^ which is approximately the Debye length of ZnO. Therefore, a high gas sensitivity can be expected from this material because metal oxide with crystal size comparable with the Debye length enable the fully electron depletion, leading to the maximum sensor response.

### 3.2. Electrical and gas sensing properties

**Fig. 5:**
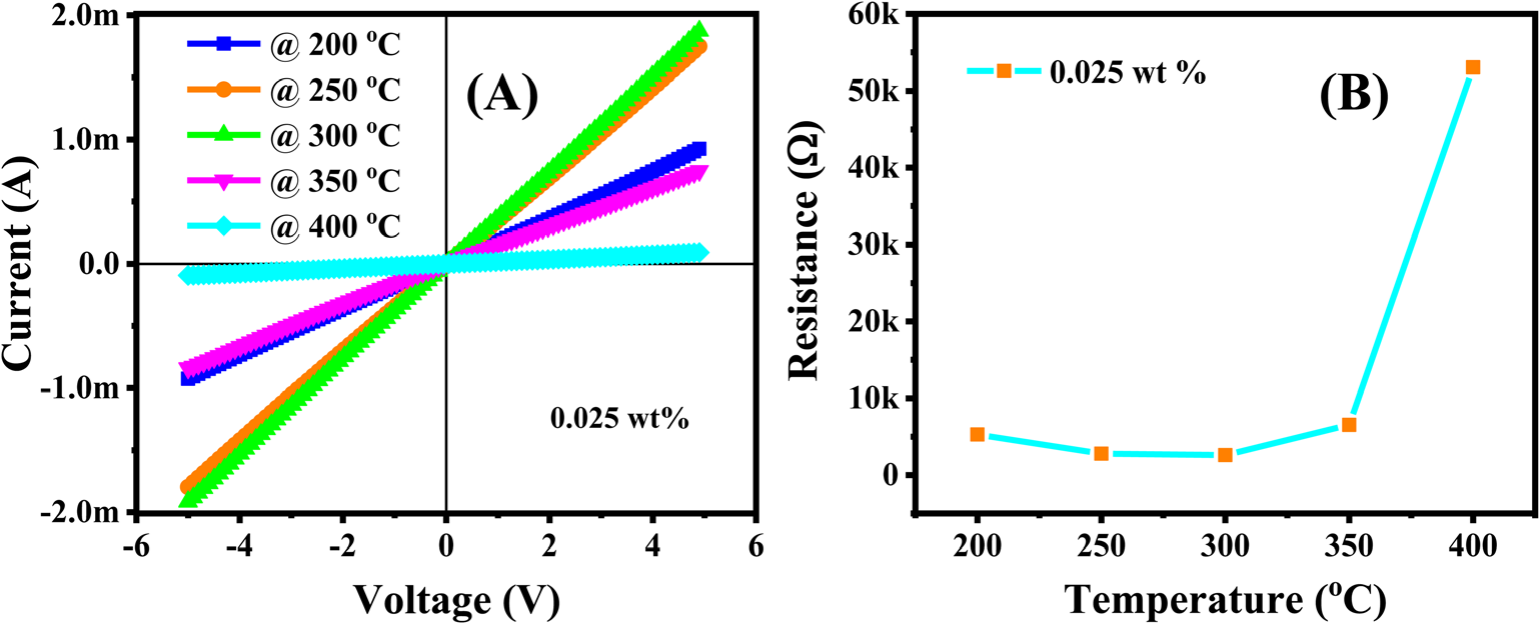
(A) The current–voltage characteristics of the Ag/Pd-doped ZnO nanoplates measured in the range of 200–400 °C in air; and (B) the calculated resistance of the sensor at different working temperatures.

Prior to the gas sensing measurements, the Ag/Pd-doped ZnO nanoplate sensors with different loading ratio (0.0125, 0.025, and 0.05 wt%) were tested for their electrical characteristics in the working temperature range by applying different bias voltages from −5 V to 5 V between the Pt electrodes. The total recorded data were ∼100 points for a given temperature with a delay time of 0.3 s between two continuous data recording by using a source meter (Keithley 2602). The *I*–*V* curves shown in Fig. 5A and S2† indicate the linear dependence of the current (*I*) on applied voltage (*V*), which is regarded as the ohmic contact between Pt and ZnO, possibly caused by the high defect level of the ZnO nanoplates.^32^ Given that the sensor was investigated at high temperatures over 100 °C, the thermal energy excited the electrons from the shallow donor region up to the conduction band, thereby decreasing the base resistance. The presence of Ag/Pd alloying nanoparticles on the semiconductor suggests the modulation of current (or resistance) with increasing temperature, as shown in Fig. 5B. In detail, the base resistance of the sensor tended to decrease with increasing temperature from 100 °C to 350 °C. Then, the resistance increased suddenly with increasing temperature over 350 °C. The two dominant conduction mechanisms are proposed to clarify this phenomenon. The variation of the sensor resistance *versus* temperature was competitive between the (i) decrease of resistance provided by thermal excitation for the semiconductor nature and the (ii) increase caused by the increase in oxygen adsorption species due to the spillover effect caused by doping metal nanoparticles. The thermal energy dominated the change in conduction at a temperature below 350 °C, while the spillover effect of the metal alloy (Ag/Pd) for enhancement of oxygen adsorption species occurred at the working temperature exceeding 350 °C.

Metal oxide semiconductor gas sensors doped with mono-metallic metals are known to show low sensing performance, limited response time as well as low selectivity toward H_2_ detection.^43^ To resolve these limitations, ultrathin ZnO nanoplates were modified on the surface by using bimetallic Ag/Pd nanoparticles. The results showed significant sensitivity at the temperature range of 200–400 °C. In this study, three different types of sensors corresponding to the different weight ratios of bimetallic Ag/Pd nanoparticles and ZnO nanoplates were tested toward H_2_ gas in the concentration range of 25–500 ppm. The sensor response to the reducing gas was determined as *S* = *R*_a_/*R*_g_. In each pulse measurement, the sensor was exposed to H_2_ for 200 s, then recovered by 400 sccm air flow until the baseline resistance was reached.

Controlling the amount of the noble metal dopant into the ZnO nanoplates that were deposited on the sensing film region is crucial to optimize the fabricated parameter and study the gas sensing features. Fig. S3A (ESI†) shows the dynamic response/recovery transient *versus* the time of the 0.05 wt% Ag/Pd–ZnO sensor obtained in the presence of various concentrations of H_2_ gas from 25 ppm to 500 ppm. The resistance of the sensor decreased and reached a saturation value upon a certain value of H_2_ exposure because of the natural behavior of an n-type semiconductor. Then, the response had a sharp drop immediately and turned to the baseline after the removal of H_2_ from the measuring chamber. The response speed was found to be slow at 200 °C with a time response of ∼190 s and decreased to 6 s when the operating temperature increased to 350 °C. This enhancement in response time was attributed to the desorption of target gas molecules and catalytic activity. The gaseous molecules became easy to remove from the surface sensing layer at higher temperature. The response value was defined at a certain H_2_ concentration, as shown in Fig. S3B (ESI†). The 0.05 wt% Ag/Pd-doped ZnO sensor exhibited a response value that tended to increase with increasing temperature. The highest response of the sensor reached 50.6 upon exposure to 500 ppm of H_2_ at 350 °C and 1.2 at 200 °C. This difference was attributed to the nature of H_2_ gas that enhanced the surface interaction at higher temperatures and numerous adsorbed oxygen species on the sensing layer generated after taking electrons from the conduction band.

**Fig. 6:**
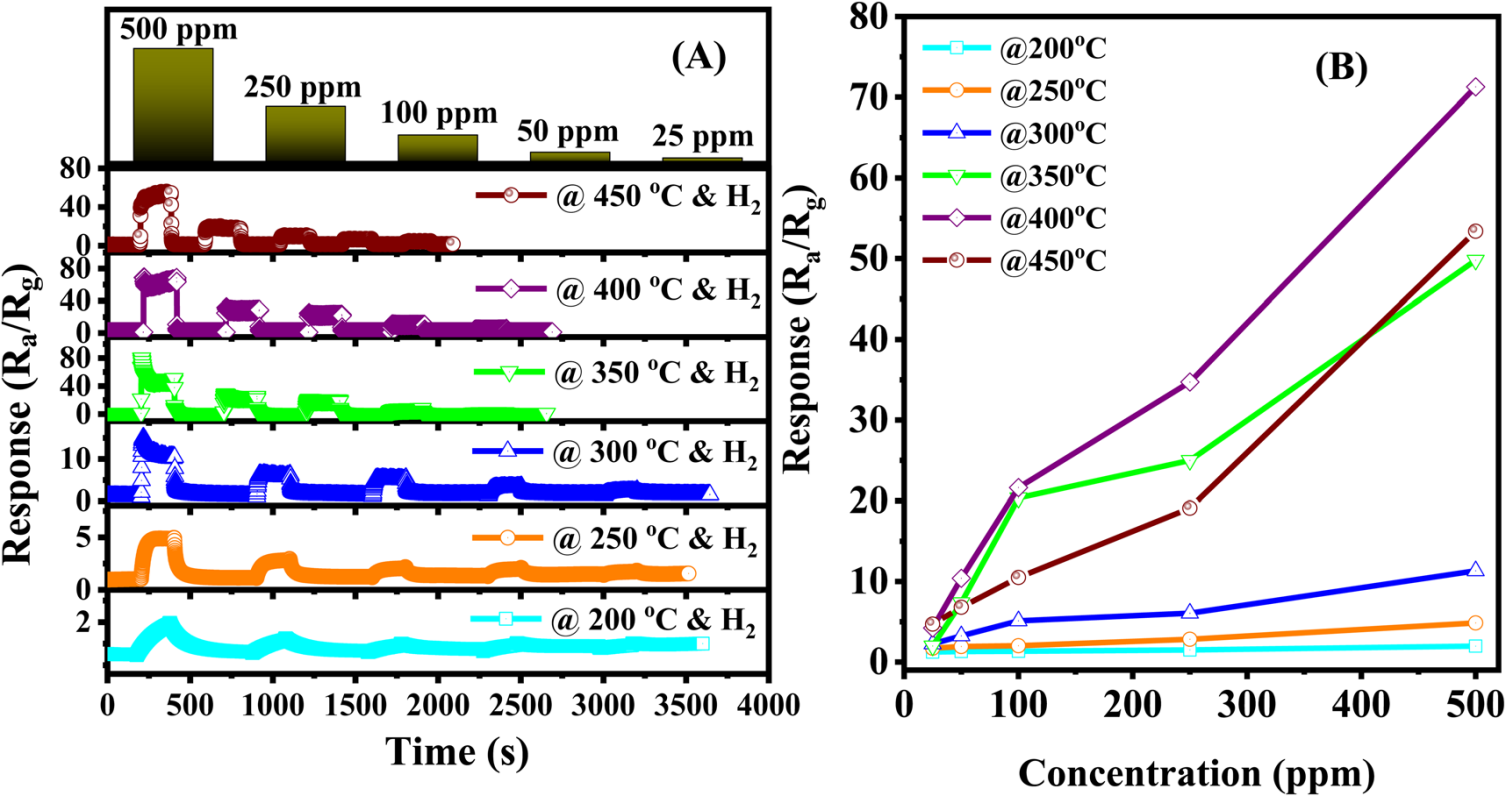
(A) The transient response curve of the 0.025 wt% Ag/Pd–ZnO nanoplate sensor towards different H_2_ concentrations in the range 200–450 °C, (B) sensor response as a function of the H_2_ concentration.

The response of the sensor improved significantly when the weight percentage of Ag/Pd nanoparticles modified on the ZnO was reduced to 0.025 wt%. Fig. 6A show the dynamic response curve of the sensor to H_2_ gas. As mentioned in the previous section, the base resistance of the sensor is changing with temperature, where the sensor resistance increased immediately in the range of 5.3–53 kΩ as the temperature increased from 200 °C to 400 °C. This result is interesting after doping the notable metal nanoparticles. The competition between two dominant conductivity mechanisms led to the unusual natural behavior of semiconductors. In all measured conditions, the sensor recover completely to the initial regime after removing H_2_ gas. Fig. 6B showed that the response value reached 78 times upon exposure to 500 ppm H_2_ at 400 °C, which was higher than that of 0.05 wt% Ag/Pd-doped ZnO sensor. Thus, the sensing performance of the sensor was enhanced remarkably after reducing the mass ratio of catalytic bimetallic.

In the case of continuous decreasing the weight percentage of the Ag/Pd alloying (*i.e.*, 0.0125 wt%), the sensor exhibited lower sensitivity compared with the 0.05 and 0.025 wt% Ag/Pd-decorated ZnO, as shown in Fig. S4A and B (ESI†). In detail, the response value reached 35 at the optimized temperature of 350 °C with a lower reactive speed. This phenomenon could be attributed to the co-catalytic phenomena and synergy effect of the bimetallic Ag/Pd in gas sensing of ZnO-based sensor tend to reduce.

**Fig. 7:**
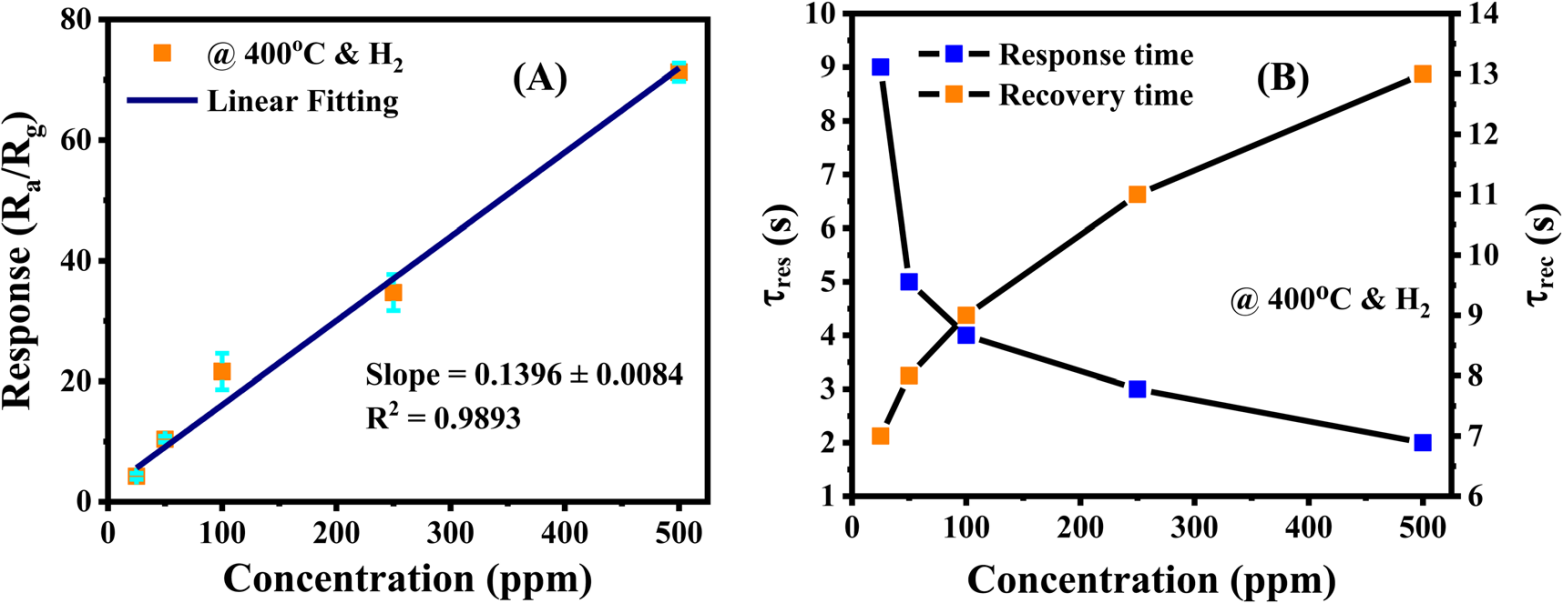
(A) The response of the gas sensor shows a linear correlation with the H_2_ gas concentrations, (B) response/recovery times as a function of the H_2_ concentration. Note that there are six trials of independent measurement were used to the error estimation in figure.

A proper calibration of the gas sensor in practical application is essential to interpolate the different concentrations in the monitoring area. As shown in Fig. 7A, the Ag/Pd-doped ZnO-based gas sensor depicted a linear fitting, the H_2_ concentration showed a first-order function behavior. The response slope was 0.1396 ppm^−1^, and the fitting quality of *R*^2^ was 0.98%. Thus, the theoretical DL of the H_2_ gas sensors was determined using the signal-to-noise ratio and the sensitivity value, which were extracted from the linear extrapolation, as follows:^44^
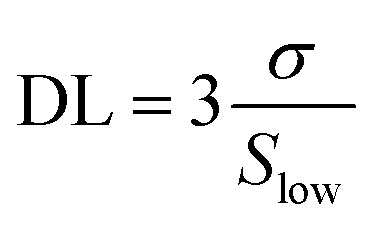
where *σ* and *S*_low_ are the standard deviation (SD) of sensor resistance in the reference gas and sensitivity at low concentration, respectively. The SD value was ∼0.04 by extracting the 50 point data before entering the H_2_ gas. The signal-to-noise was calculated at the steady-state operating conditions, which was estimated to be 36 in this sensor. Hence, the DL of the proposed H_2_ gas sensor at 400 °C is approximately 800 ppb. Note that in the measured range (25–500 ppm), the sensor showed linger dependent of response on H_2_ concentration without signal of saturation, thus the sensor has potential to detect H_2_ higher than 500 ppm.

The response and recovery times of the sensor were determined from the response curve *versus* reaction time; that is, its value reached 90% saturated resistance. Fig. 7B shows the response and recovery times of the Ag/Pd(0.025 wt%)-doped ZnO nanoplate sensor, which were calculated at 400 °C at different H_2_ concentrations. The sensor indicated ultrafast reaction speed, and its value is of several seconds. For instance, the response and recovery times for the detection of 500 ppm H_2_ gas were 2 s and 13 s, respectively. The response time increased with the decrease in introduced concentration. By contrast, recovery time would be reduced upon exposure to lower H_2_ concentration. This phenomenon was due to the adsorption and desorption gaseous mechanism. That is, the target molecules were easily transported into the surface of sensing material at high H_2_ gas concentration. However, the target molecules were desorbed easily from the surface of the sensing layer at low concentration after being refreshed by airflow. The results are in good agreement with other reports.^44,45^

**Fig. 8:**
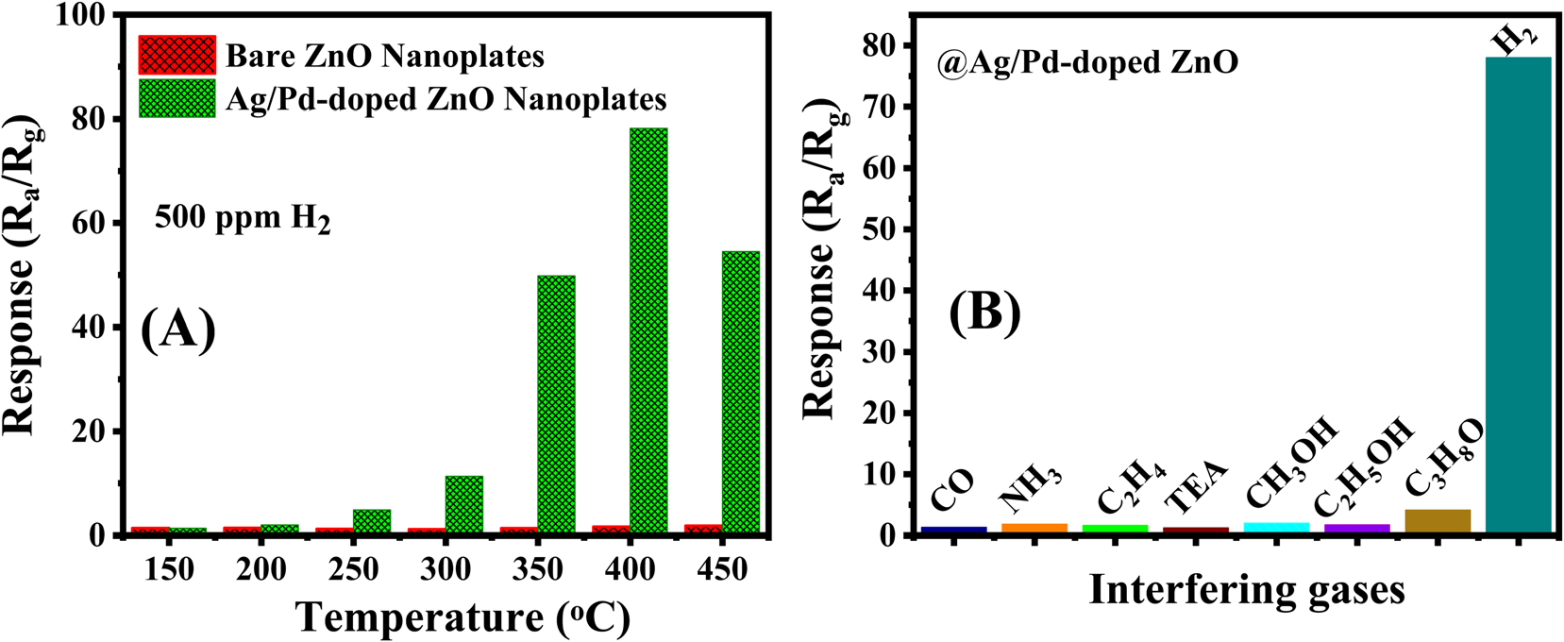
(A) Comparison in the responses of the sensors based on pristine ZnO and 0.025 wt% Ag/Pd–ZnO toward 500 ppm H_2_ at different temperatures and the (B) selectivity of the Ag/Pd-doped ZnO sensor in the presence of interfering gases.

To demonstrate the enhancement in H_2_ sensing performance after modifying the Ag/Pd nanoparticles, the pure ZnO nanoplates and the Ag/Pd-doped ZnO nanoplate-based sensors were tested toward the detection of 500 ppm H_2_ in the temperature range of 150–450 °C. As shown in Fig. 8A, the Ag/Pd-doped ZnO nanoplate sensor exhibited superior gas sensing performance with a response value of 47-fold larger than that of the pure ZnO counterpart at its optimized temperature.

To evaluate the selectivity of the H_2_ gas sensor, the Ag/Pd-doped ZnO sensor was measured to various interfering gases, namely, CO, NH_3_, C_2_H_4_, and VOCs (triethylamine, ethanol, methanol, and acetone), under concentration of 500 ppm at 400 °C. As seen in Fig. 8B, the sensor exhibited an excellent response to H_2_ compared with those to other gases due to the unique interaction between the notable metal and H_2_ gas molecules during the adsorption process. This result suggested that the Ag/Pd-doped ZnO nanoplate-based sensor is a potential candidate for monitoring and/or alarming H_2_ leakage over the contamination with other gases.

**Fig. 9:**
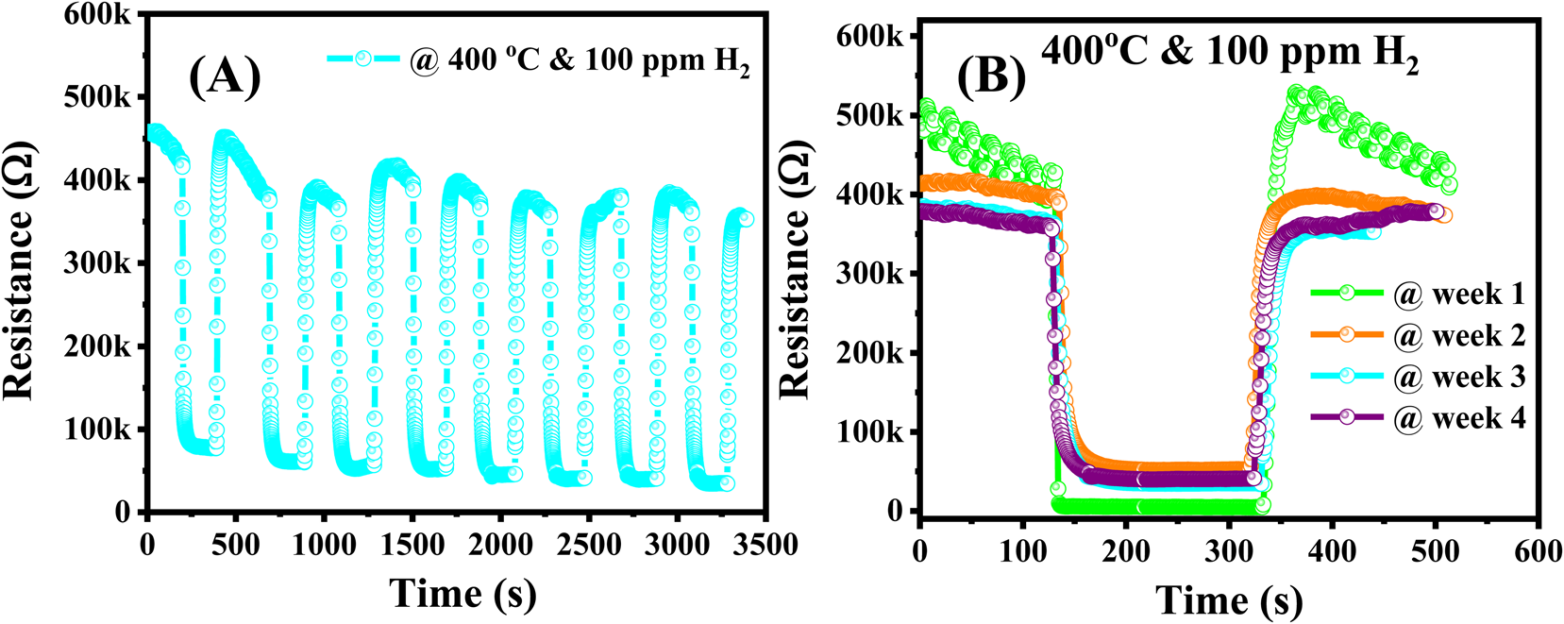
(A) The short-term and (B) long-term stability of the 0.025 wt% Ag/Pd–ZnO sensor after 4 continuous weeks of testing at 400 °C.

In addition to the short-term stability was evaluated by testing the sensor over eight cycles on/off of 100 ppm of H_2_ gas. No obvious decay in response was observed during the testing process as shown in Fig. 9A, indicating a fascinating repeatability in terms of response and recovery capability. The long-term stability was also tested continuously to 100 ppm of H_2_ in four weeks. As shown in Fig. 9B, the dynamic resistance had good long-term stability with small variation in response values for a week of testing.

**Fig. 10:**
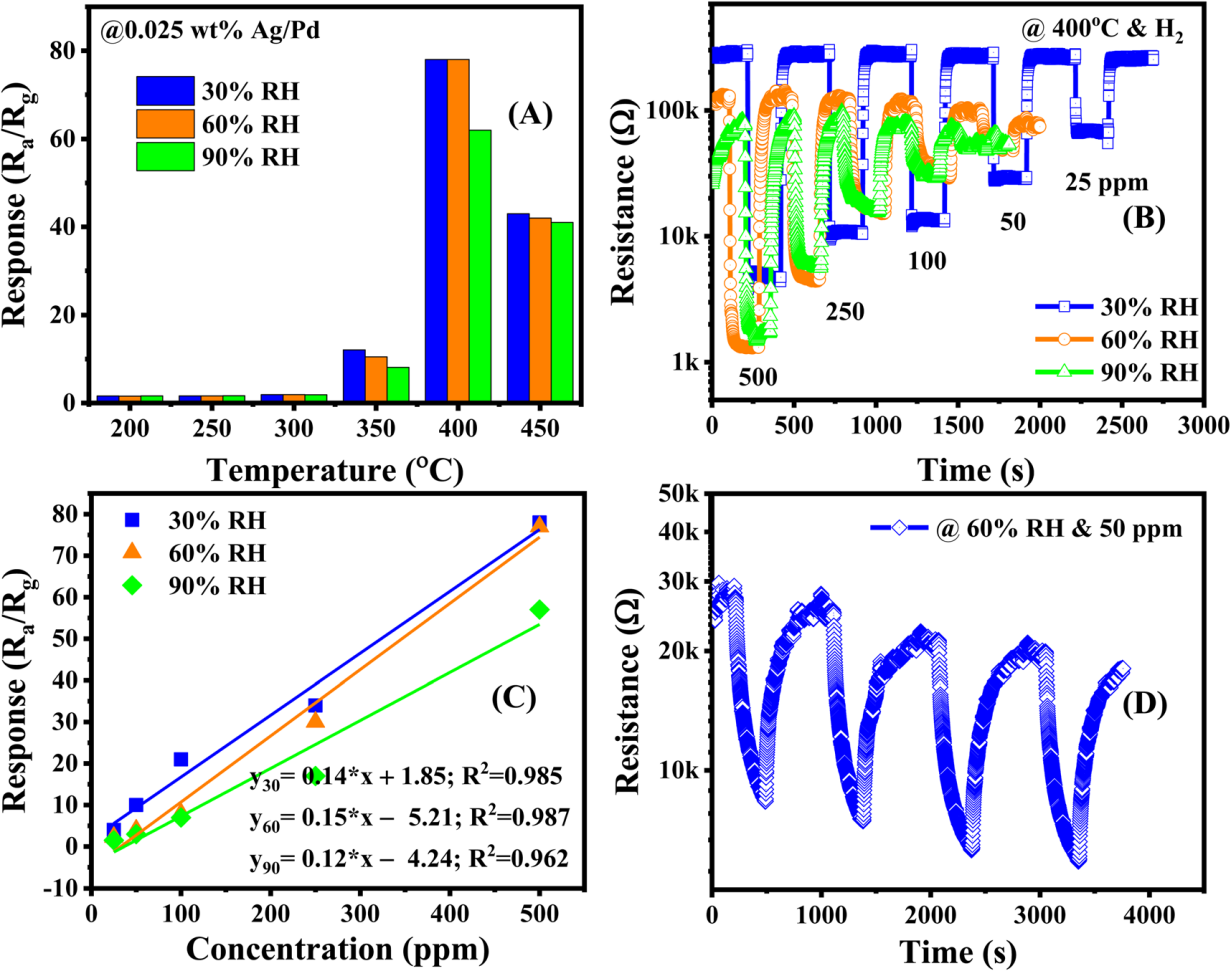
(A) The response in the temperature range of 200–450 °C (B) the dynamic resistance *versus* time, (C) calibration curve under various relative humidity, and (D) short-term stability toward 50 ppm H_2_ concentration in the presence of 60% RH of the 0.025 wt% Ag/Pd–ZnO sensor.

The previously discussed gas sensor behavior of Ag/Pd-doped ZnO toward H_2_ was observed under dry air conditions. Nevertheless, in practical applications, such as storage and transport places, the presence of humidity in the atmosphere results in the noise signal at the different periods of recorded data. To outstanding the behavior of humid condition to the dynamic response of sensor, the Ag/Pd–ZnO based sensor is investigated in the presence of water vapor. Fig. 10A show the response of the 0.025 wt% Ag/Pd–ZnO based sensor toward 500 ppm H_2_ in the temperature range of 200–450 °C with various relative humidity. There is marginal fluctuation in response when the sensor operates in the high humid condition (*i.e.*, 90% RH). The decrease in response was attributed to the water molecules adsorbed on the surface which prevented numerous H_2_ gas molecules to be adsorbed and react with previously generated oxygen species. The dynamic resistance of sensor in the range H_2_ concentration of 25–500 ppm were given in Fig. 10B. The sensor recovered completely to initial baseline; however, it is getting slow reaction with H_2_ gas molecules when increasing the humidity level. Additionally, the base resistance of the Ag/Pd-doped ZnO sensor decreased with increasing relative humidity. In terms of the pure ZnO material under humid conditions, the OH^−^ group comprehensively adsorbed on the ZnO surfaces and O^−^ species adsorption was perturbed by the presence of OH^−^. The water molecules operated as the electron acceptor, resulting in a decrease in the number of electrons in ZnO materials. Otherwise, the resistance of the pure ZnO sensor tended to increase. However, when decorated with Ag/Pd nanoparticles, the adsorption of O^−2^ species on the metal catalyst prevented OH^−^. This phenomenon reduced the number of the extracted electron from the conduction band caused by the water molecules.^46^ The calibration curves were extracted from resistance response graph and fitted with first-order equation at 30, 60 and 90% RH at 400 °C (Fig. 10C). The figure shows good linear characteristic between H_2_ concentration and sensor response. Besides, the sensitivity of sensor is estimated approximately 0.14, 0.15, and 0.12 ppm^−1^, corresponding with 30, 60, and 90% RH. Inspire of working in the humid circumstance, the sensor was subjected to seven consecutive cycles of 50 ppm H_2_, the data is shown in Fig. 10D. The graph shows that the sensor reaches fascinating repeatability and small drift.

Table 1 shows a comparison of recent reports in the scientific literature with conductive sensors based on pristine, composite, and modified notable metal (*i.e.*, Ag, Pd, Pt, and Co) ZnO nanomaterial. ZnO-based resistive sensors were grown by different methods to achieve various morphologies. Such morphologies exhibited different response characteristics in H_2_ gas monitoring. Similar to other metal oxides, ZnO-based sensors are almost applied in detecting H_2_ gas at high temperatures, that is, in the range of 200–450 °C with a slightly small response value and slow reaction speed. As illustrated in Fig. S5 (ESI†), the response and recovery time of the Ag/Pd–ZnO sensor are approximately 2 s and 13 s; much faster than those of the pure ZnO sensor of 77 s and 78 s, respectively. Here, the Ag/Pd–ZnO sensor is high competition with other sensors in terms of high response value, detection limit, and response/recovery speed.

**Table 1:** Comparison of ZnO nanostructure-based gas sensors for detecting hydrogen gas in recent scientific literature

Material	Method	Concentration (ppm)	Response	*τ*_res_/*τ*_rec_ (s)	Temperature (°C)	LOD (ppm)	Ref.
Rose-shaped ZnO	Hydrothermal	50 (10–150)	50^b^	360/960	270	0.01	47
Pt-decorated ZnO NPs	Magnetron sputtering	1000 (200–1000)	14.9^b^	133/112	300	100	48
ZnO/Pt thin films	RF sputtering	15 (0–15)	6.3^a^	—	250	0.15	21
Pt-decorated ZnO	RF sputtering	1200 (75–1200)	94^b^	240/500	300	75	49
Pd capped ZnO NRs	Sputtering	500 (100–1000)	3.6^b^	59/51	350	100	50
Pd-functionalized ZnO nanowires	Vapor–liquid–solid technique	100 (0.1–100)	87.17^a^	—	350	0.1	51
Pd–ZnO nanosheet	Solvothermal method	50 (0.1–10)	2.514 a	336/294	250	0.5	22
Spherical Au on ZnO thin films	RF magnetron sputtering	50 (50–1000)	79^b^	25/55	250	100	52
In-doped ZnO thin film	Chemical solution deposition	5 (1–1660)	15^b^	—	300	1	53
Ag/ZnO hollow microstructures	Chemically coprecipitation	300 (5–300)	479^b^	175/655	250	5	23
Co_3_O_4_-loaded ZnO nanofibers	Electrospinning	10 (1–10)	133^b^	—	300	1	54
ZnO/Co	Hydrothermal	3000 (1000–3000)	97^a^	74/40	300	1000	55
**0.025 wt% Ag/Pd-doped ZnO**	**Hydrothermal combine polyol**	**500 (25–500)**	**78** a	**2**/**13**	**400**	**0.8**	**This study**

a Response defined as *R*_a_/*R*_g_.

b Response defined as (*R*_air_ − *R*_gas_)/*R*_gas_ ×100 (%).

The superior hydrogen sensing properties of the Ag/Pd-doped ZnO sensors with respect to the pristine ZnO could be attributed to many factors as follows: the change in the barrier height *via* charge transfer between Ag/Pd alloy and the ZnO surface; the spillover effect in enhancing the preabsorbed oxygen species; and the catalytic effect of Ag/Pd alloying nanoparticles in dissociation of H_2_ molecule.

**Fig. 11:**
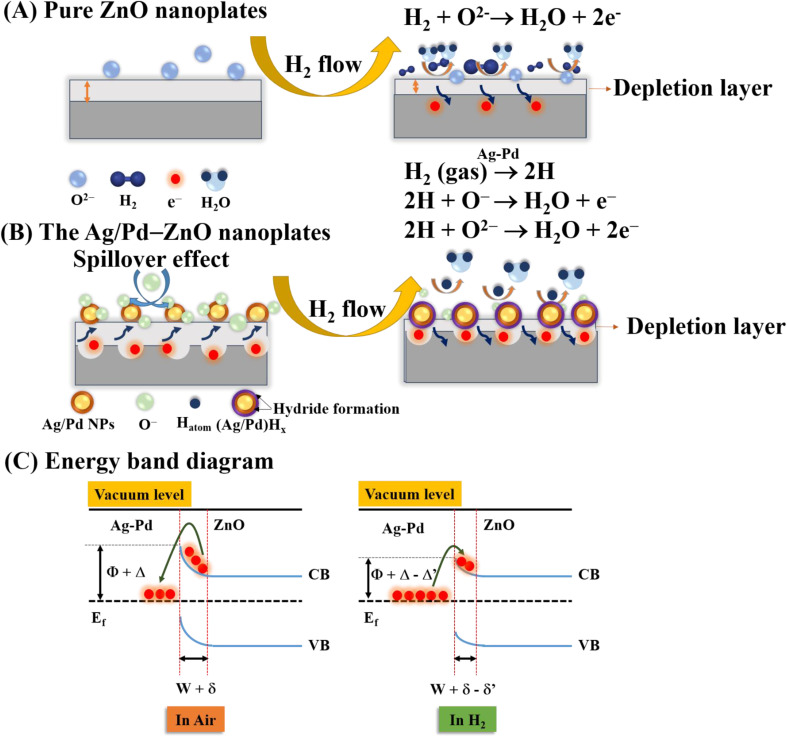
Schematic for the H_2_ gas sensing mechanism of (A) pristine ZnO, (B) AgPd-doped ZnO, and (C) the energy band diagram for the Ag/Pd-doped ZnO in the presence of air and H_2_ (*Φ*, *Δ*, and *Δ*′ denote the barrier height at the interface Ag–Pd NPs and ZnO and the modified barrier heights in air and H_2_ gas, respectively; *W*, *δ*, and *δ*′ are the depletion layer width and the modified depletion layer widths in presence of air and H_2_, respectively).

To understand the influence of bimetallic nanoparticles on the gas sensing mechanism, the chemical adsorption activities and energy band diagram of pure ZnO and the Ag/Pd–ZnO nanoplates in the air and H_2_ gas are proposed (Fig. 11). When the sensor was placed in the air, the oxygen molecules were adsorbed onto the surface of ZnO nanoplates and captured the free electrons to generate the oxygen species (eqn (1) and (2)), resulting in the formation of depletion layer with low conductivity near the surface (Fig. 11A).

1 O_2(gas)_ + 2e^−^ → O^−^_2(ads)_

2 O_2(gas)_ + 2e^−^ → 2O^−^_(ads)_

Upon exposure to H_2_ gas, the H_2_ molecule react with pre adsorbed oxygen species and releases free electrons back to the conduction band of ZnO, as eqn (3) and (4). As a result, the release of free electrons decreases the resistance of bare ZnO sensor.

3 H_2_ + O^−^ → H_2_O + e^−^

4 H_2_ + O^2−^ → H_2_O + 2e^−^

However, when the ZnO material was functionalized by noble metal, the spillover effect was considered. In this circumstance, the introduced gas was adsorbed on the surface of the bimetallic Ag/Pd NPs. Afterward, it dissociated and migrated to the sensing material, thus enhancing the preabsorbed oxygen species. The gas adsorption rate before and after doping the noble metal are demonstrated in (Fig. 11B). A larger number of oxygen species was generated on the surface of the Ag/Pd-doped ZnO sensor compared with pure ZnO, leading to an expansion in the space-charge region in the doped ZnO material. That is, the baseline electrical resistance of doped ZnO in air became higher than that of pristine ZnO. Once the Ag/Pd-doped ZnO sensor was exposed to H_2_, the H_2_ molecules reacted with the preabsorbed oxygen species to form water and release free electron, as following eqn (5)–(7):^56^5
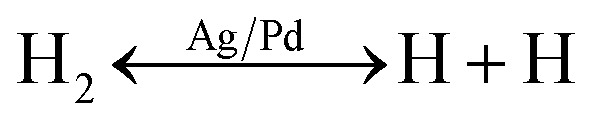

6 2H + O^−^ → H_2_O + e^−^

7 2H + O^2−^ → H_2_O + 2e^−^

Numerous free electrons returned to the conduction band of ZnO, thereby reducing the electron depletion layer width. Thus, the resistance of the sensor decreased. For the Ag/Pd–ZnO nanoplate sample, when the H_2_ molecule was introduced, the sample was split into two active atoms (*i.e.*, H atoms) due to the spillover effect of the Ag/Pd nanoparticles, according to eqn (5).^57^ Then, these atoms with high mobility easily reacted with the preabsorbed oxygen species, thereby enhancing the response, according to eqn (6) and (7).

In addition, the formation of the barrier height at the interface of the Ag/Pd nanoparticles and the surface of ZnO also contributed to the enhancement of gas sensing properties. The electron transfer from the ZnO nanomaterial to Ag/Pd was clearly bent at the interface between ZnO and Ag/Pd alloy due to the difference in work function (*Φ*_ZnO_ = 4.75 eV, *Φ*_Pd_ = 5.1 eV, and *Φ*_Ag_ = 4.9 eV). Thus, the barrier potential of the doped ZnO became higher than that of the undoped one, as shown in (Fig. 11C). When H_2_ gas was injected into the sensor, the barrier height tended to decrease, and the depletion layer width became smaller than that in air. However, when Ag/Pd nanoparticles were decorated on the ZnO surface, H_2_ molecules adsorbed on the bimetallic surface would be dissociated and chemisorbed into the metal hydride form. This metal hydride had a lower work function compared with ZnO, resulting in the transfer of electrons from the metal hydride to ZnO bulk. This phenomenon led to the return of numerous electron carriers to the conduction band of ZnO, resulting in a decrease in the resistance of the sensor.

## 4. Conclusion

Ultrafine ZnO nanoplates were functionalized with Ag/Pd alloying nanoparticles to enhance the hydrogen gas sensing performance. The ZnO nanoplates were prepared *via* the hydrothermal method, following heat treatment at 600 °C in air. The Ag/Pd alloying nanoparticles with an average diameter of 8 nm were prepared using a modified polyol method. Different amounts of Ag/Pd nanoparticles were decorated on the surface of the ZnO nanoplates to enhance the gas sensing properties. The results showed that the 0.025 wt% Ag/Pd–ZnO nanoplate sensor exhibited superior gas sensing performance compared with the pure ZnO counterpart. The Ag/Pd-doped ZnO sensor exhibited 48-fold higher response than that of the pure ZnO sensor upon exposure to 500 ppm of H_2_ gas at optimized temperature. Furthermore, the Ag/Pd-doped ZnO sensor exhibited high speed response, high selectivity, and good repeatability. The incorporation of Ag/Pd nanoparticles into the ZnO nanoplates produced considerable oxygen active sites and improved the adsorption of H_2_ gas because of the catalytic nature of Ag/Pd nanoparticles. Thus, the Ag/Pd-doped ZnO sensor is an excellent candidate for detecting H_2_ gas leakage in next generation of hydrogen energy devices because of its outstanding performance and facile synthesis.

## Conflicts of interest

There are no conflicts to declare.

## Supplementary Material

RA-013-D3RA01436C-s001
