# Supplementary material for: A sigh-performance hydrogen gas sensor based on Ag/Pd nanoparticle-functionalized ZnO nanoplates
Source: RSC Adv. 2023 Apr 26;13(19):13017–29. doi: 10.1039/d3ra01436c (PMC10132452; doi:10.1039/d3ra01436c)
Supplement: RA-013-D3RA01436C-s001 [file RA-013-D3RA01436C-s001.pdf]

Electronic Supplementary Material (ESI) for RSC Advances.
This journal is © The Royal Society of Chemistry 2023

Electronic Supplementary

High-performance hydrogen gas sensor based on Ag/Pd nanoparticles functionalized ZnO nanoplates

To Thi Nguyet[1], Dang Thi Thanh Le[1], Nguyen Van Duy[1], Chu Thi Xuan[1], Sven Ingebrandt[2], Xuan Thang Vu[2], Nguyen Duc Hoa[1*]

[1] International Training Institute for Materials Science (ITIMS), Hanoi University of Science and Technology (HUST);

[2] Institute of Materials in Electrical Engineering 1, RWTH Aachen University, Sommerfeldstr. 24, 52074 Aachen, Germany

Corresponding author: Email: ndhoa@itims.edu.vn /hoa.nguyenduc@hust.edu.vn

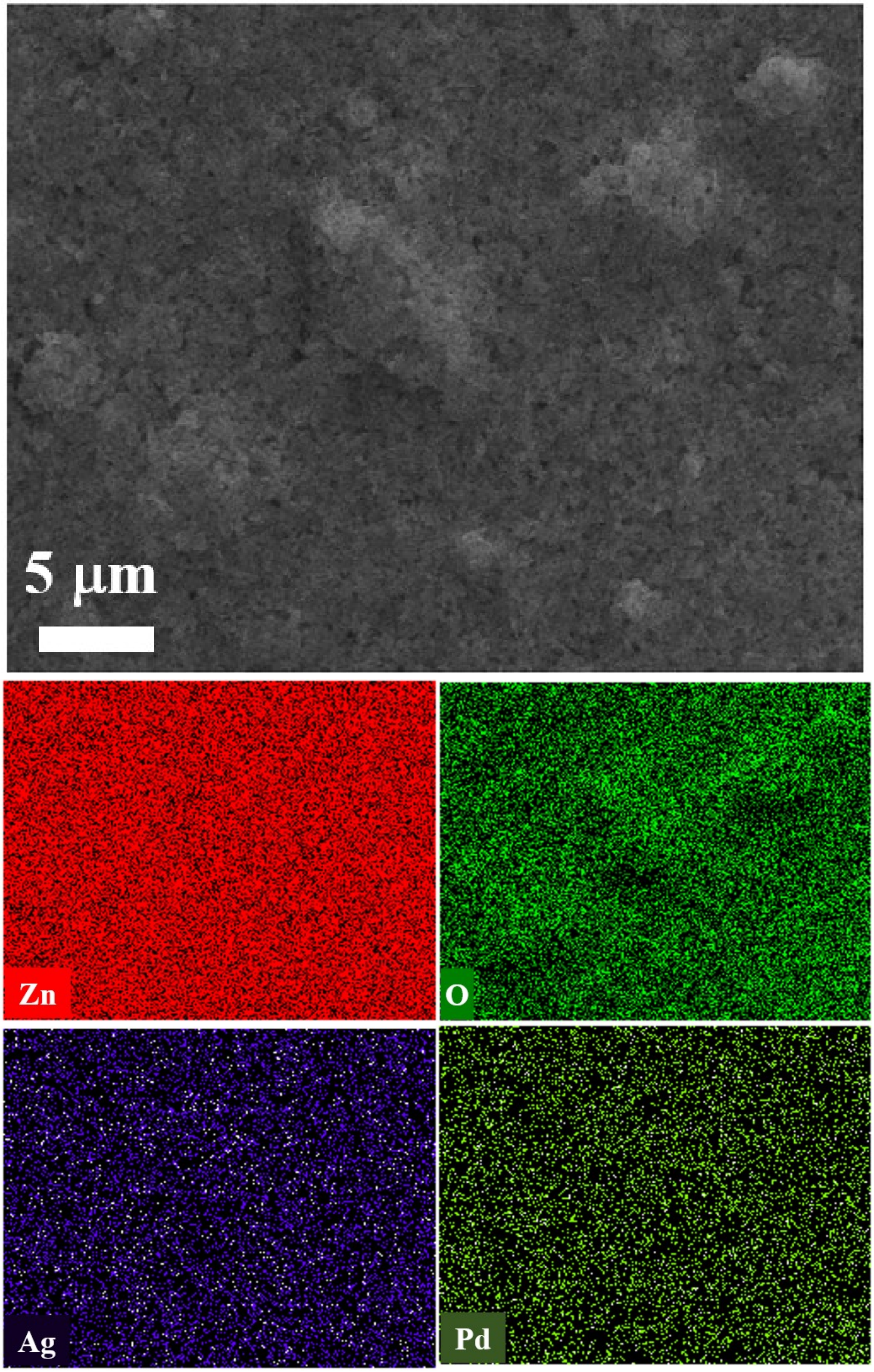

Figure S1. *SEM image, and EDS mapping of the 0.25 wt% Ag/Pd-doped ZnO sample*

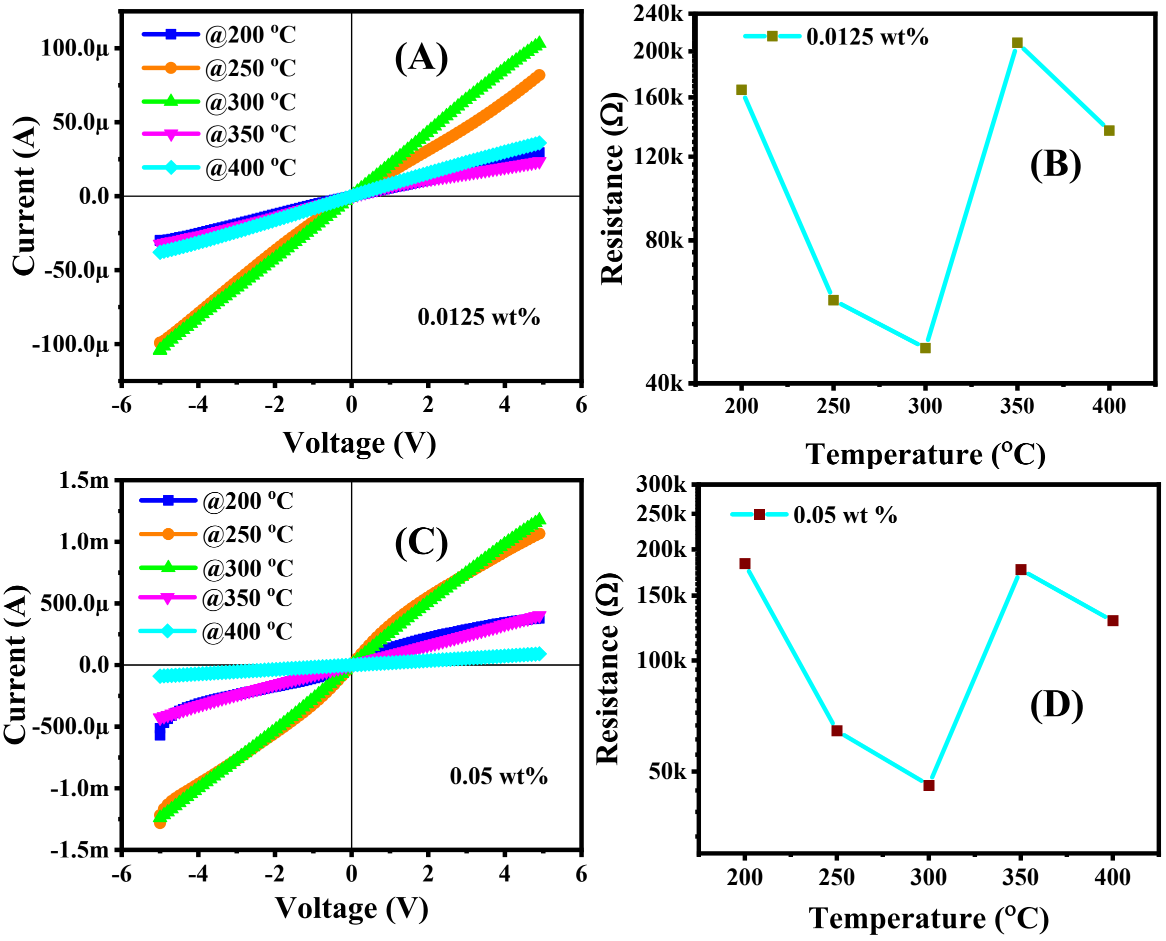

Figure S2. *The I-V curves and calculated resistance of (A-B) the 0.0125 wt% and 0.05 wt% (C-D) Ag/Pd-doped ZnO measured in the range of 200 – 400°C*

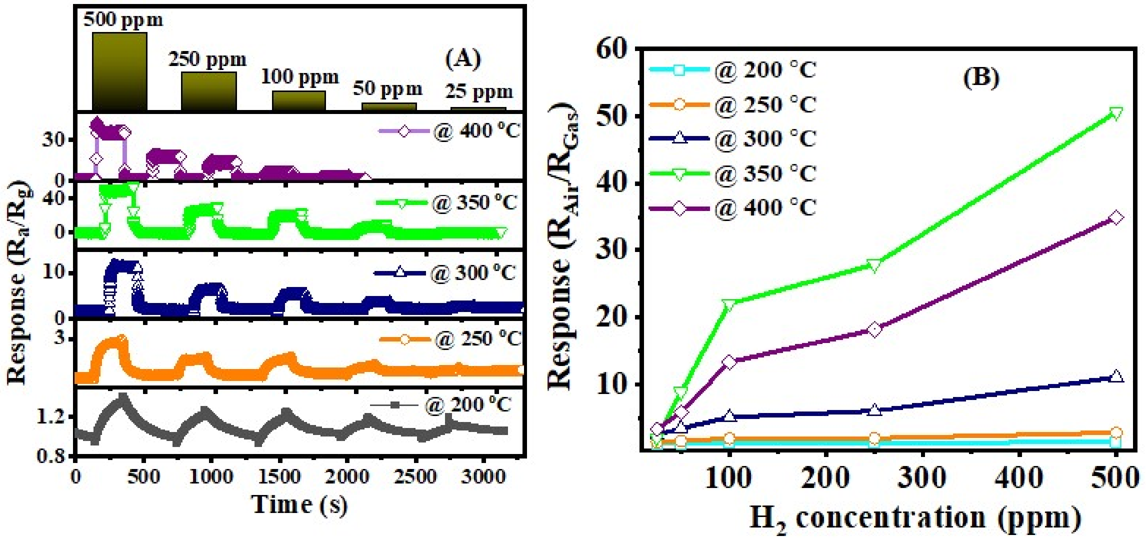

Figure S3. *(A) The transient response curve of the 0.05 wt% Ag/Pd-ZnO sensor towards different H_2 concentrations in the range 200–400 °C; (B) sensor response as a function of the H_2 concentrations*

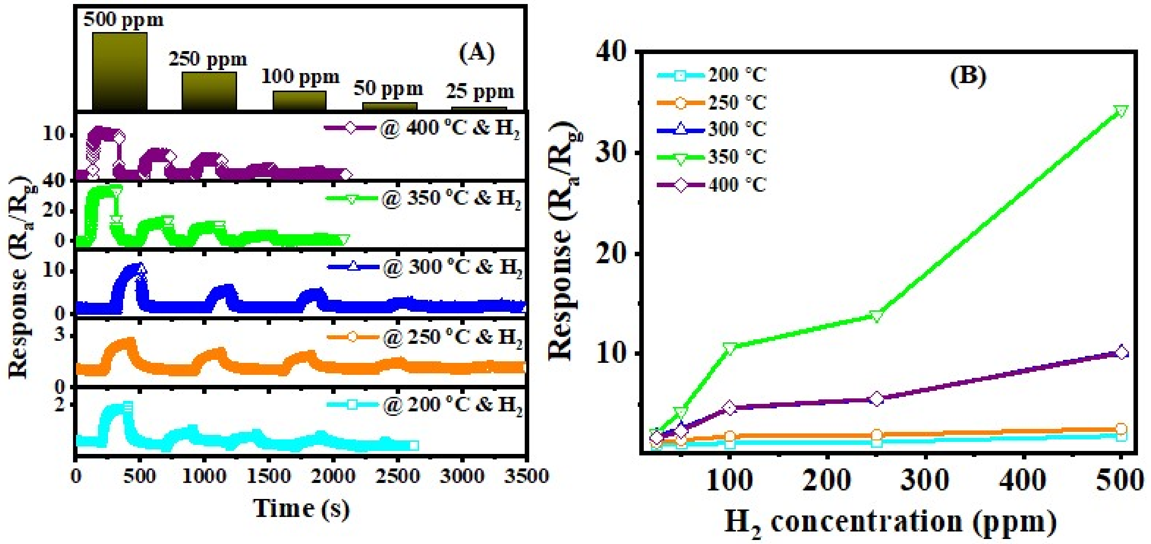

Figure S4. *(A) Transient response curve of the 0.0125 wt% Ag/Pd-ZnO sensor toward different H_2 concentrations in the temperature range of 200–400 °C; (B) sensor response as a function of the H_2 concentration*

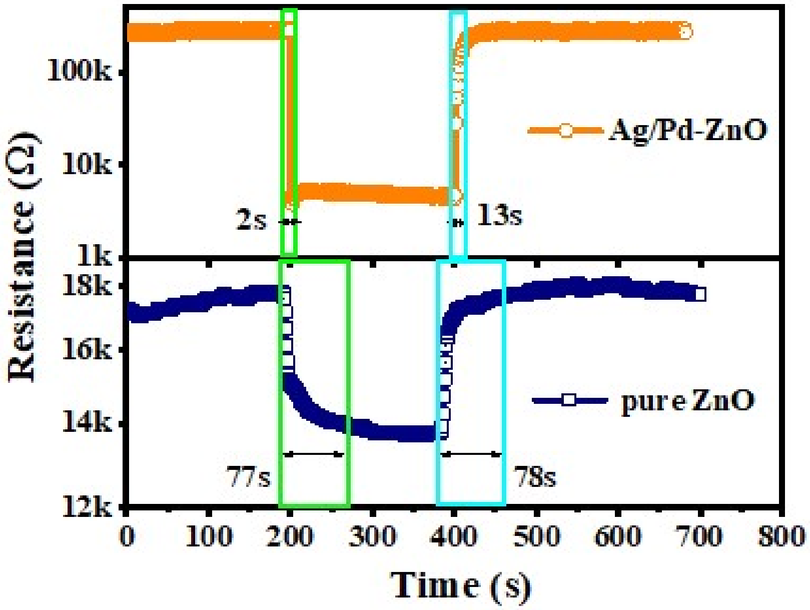

Figure S5. *Comparison in the dynamic response of pure ZnO and 0.25 wt% Ag/Pd-ZnO sensor toward 400 ^{o}C*

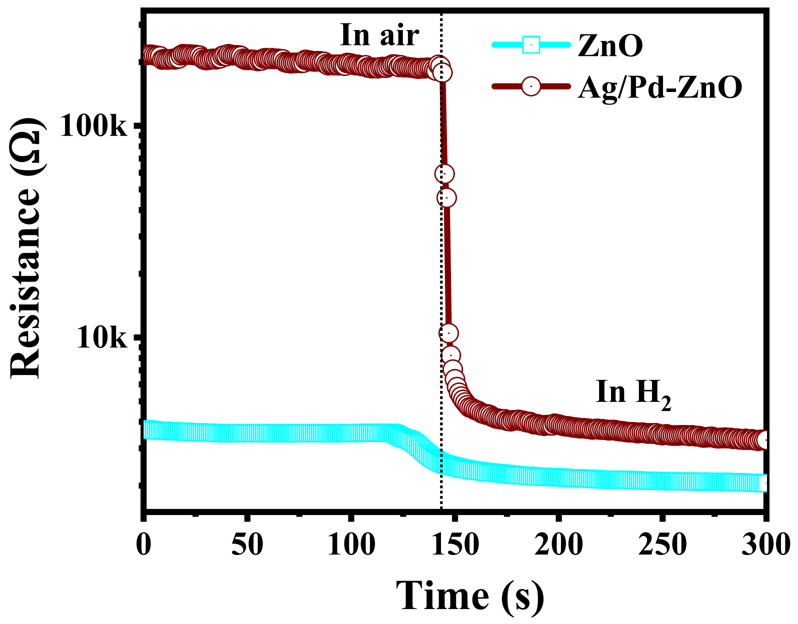

Figure S6. *The resistance curve of the pristine ZnO and 0.25 wt% Ag/Pd-doped ZnO sensor in air and H_2 gas at 400^oC*
